# Quantitative Thermal Testing Profiles as a Predictor of Treatment Response to Topical Capsaicin in Patients with Localized Neuropathic Pain

**DOI:** 10.1155/2017/7425907

**Published:** 2017-02-21

**Authors:** A. Serrano, D. Torres, M. Veciana, C. Caro, J. Montero, V. Mayoral

**Affiliations:** ^1^Department of Anesthesiology, Critical Care and Pain Clinics, Hospital Universitari de Bellvitge, L'Hospitalet de Llobregat, 08907 Barcelona, Spain; ^2^Department of Anesthesiology, Critical Care and Pain Clinics, Hospital Universitario de Santiago de Compostela, 15701 Santiago, Spain; ^3^Department of Neurology and Neurophysiology, Hospital Universitari de Bellvitge, L'Hospitalet de Llobregat, 08907 Barcelona, Spain

## Abstract

There are no reliable predictors of response to treatment with capsaicin. Given that capsaicin application causes heat sensation, differences in quantitative thermal testing (QTT) profiles may predict treatment response. The aim of this study was to determine whether different QTT profiles could predict treatment outcomes in patients with localized peripheral neuropathic pain (PeLNP). We obtained from medical records QTT results and treatment outcomes of 55 patients treated between 2010 and 2013. Warm sensation threshold (WST) and heat pain threshold (HPT) values were assessed at baseline at the treatment site and in the asymptomatic, contralateral area. Responders were defined as those who achieved a > 30% decrease in pain lasting > 30 days. Two distinct groups were identified based on differences in QTT profiles. Most patients (27/31; 87.1%) with a homogenous profile were nonresponders. By contrast, more than half of the patients (13/24, 54.2%) with a nonhomogenous profile were responders (*p* = 0.0028). A nonhomogenous QTT profile appears to be predictive of response to capsaicin. We hypothesize patients with a partial loss of cutaneous nerve fibers or receptors are more likely to respond. By contrast, when severe nerve damage or normal cutaneous sensations are present, the pain is likely due to central sensitization and thus not responsive to capsaicin. Prospective studies with larger patient samples are needed to confirm this hypothesis.

## 1. Introduction

Neuropathic pain is a chronic condition that is very difficult to treat [1], and only small improvements in treatment efficacy have been achieved in the last decade. However, one of the most promising treatments for peripheral neuropathic pain (PeNP) developed in recent years is the capsaicin 8% patch (CP8%) (QutenzaTM). This patch delivers capsaicin into the skin and clinical trials have shown that it can provide up to 12 weeks of PeNP relief with a single topical patch application [2–4].

Capsaicin works by directly targeting, with a highly selective agonism, the transient receptor potential vanilloid 1 (TRPV1) receptor, which is primarily present in C-fibers and in some A*δ*-fibers. High-concentration capsaicin activates TRPV1 channels by overstimulating the nociceptors, resulting in the defunctionalization of the nociceptor nerve fibers and thereby reducing spontaneous nerve activity, leading to a loss of responsiveness. After defunctionalization, patients perceive a decrease in PeNP [5–7], which is frequently referred to as “desensitization.”

The European Medical Agency (EMA) has recommended that CP8% be applied by a doctor or other healthcare professionals under the supervision of a doctor [8]. However, this recommendation limits treatment options, primarily because it depends on the availability of treatment at outpatient clinics. This requirement also makes treatment more expensive. In addition, the indirect costs of personnel and other materials must be added to the direct cost of CP8% [9]. As a result, many patients who could benefit from treatment may not be treated due to affordability issues. The number-needed-to-treat (NNT) for CP8% is high [10]. Given the aforementioned high cost of treatment, the patch should be applied only to those most likely to benefit from improvement. However, to date, no clear predictors of treatment response in patients with PeNP have been identified, despite recent efforts to do so [11–13].

Given that capsaicin affects unmyelinated or slightly myelinated fibers and studies have shown that the CP8% patch involves heat sensation [6], we hypothesized that quantitative thermal testing (QTT) could be a potential predictor of treatment response. To investigate this question, we performed a retrospective study of clinical records to assess the QTT profiles of patients who underwent capsaicin treatment for PeLNP. Our main aim was to compare the baseline QTT profiles to determine whether any of these were associated with treatment response.

## 2. Material and Methods

This was a retrospective analysis of all patients treated with the CP8% patch for PeNP at our pain clinic between 2010 and 2013. From patient medical records, we obtained QTT results and treatment outcomes (response versus nonresponse) for all patients. All procedures were performed according to clinical protocols in place at our institution during the study period.

### 2.1. Patients

Since late 2010, all patients presenting at our pain clinic with a high suspicion for PeNP have been asked to complete the* Douleur Neuropathique *4 Scale (DN4), followed by consultations with a pain physician and a neurophysiologist for complete neurological assessment. Based on this evaluation, patients are classified into one of the following pain categories: (1) postherpetic neuralgia (PHN) and (2) chronic postsurgical pain (CPSP), or type I or II complex regional pain syndrome (CRPS). PeNP is considered possible when the patient meets the criteria given in the International Association for the Study of Pain (IASP) recommendations [14]. In cases in which the PeNP is confined to a single, localized area, it is classified as localized peripheral neuropathic pain (PeLNP) [15]. Patients with PeLNP are eligible to choose either CP8% or lidocaine plaster for topical treatment.

### 2.2. Establishing the Area to Be Treated

Prior to treatment, a discontinuous line is drawn with a permanent-ink marker on the skin to mark the boundaries between the painful area (identified by the patient as hyperalgesia or allodynia) and the area with normal skin sensation. Transparent paper is used to copy this area and save for further patch applications.

### 2.3. Assessment of Pain

A numerical pain rating scale (NPRS), ranging from 0 to 10 points, is used to assess the pain level, with 0 indicating no pain and 10 indicating the worst imaginable pain. Patients are asked to rate their pain scores at the following times: at first visit, before patch application, throughout the treatment procedure (see Section 2.6), at one week after application, and at 4–6 weeks after treatment. One week after treatment, patients are contacted by telephone to assess discomfort, heat pain, and any analgesics used.

### 2.4. Quantitative Thermal Testing

A QTT profile is routinely obtained in all patients at the following time points: pretreatment, one to two weeks after the first consultation and at PeNP diagnosis. The QTT test is always performed in the same room under the same environmental conditions. We determine warm sensation threshold (WST) and heat pain threshold (HPT) values, first in the normal contralateral skin area and then in the affected skin area, using the limits method [16]. The QTT test is performed using the pathway 9.0 cm^2^ thermode (MEDOC Ltd., Israel) [17, 18]. The baseline temperature is set at 32°C (center of neutral range), with a cut-off temperature of 50°C and a ramp rate of 1°C/s for warm threshold and 1.5°C/s for heat pain. Patients are instructed to press a button (which returns the temperature to baseline) as soon as they perceive a warm or heat pain sensation. For each threshold, the test is repeated from 3 (minimum) to 5 (maximum) times. Based on these tests, a mean threshold value and standard deviation (SD) are automatically calculated.

### 2.5. Treatment with Capsaicin 8% Patch

On the day of treatment, the patients are told to eat a small meal (either breakfast or lunch, as appropriate) before presenting at the outpatient clinic. Topical local anaesthetic is applied for 75–90 minutes before procedure. The anaesthetic cream is then removed and the skin washed and dried. Afterwards, the patch is cut to fit using the previously created transparent sheet and applied to the patient's skin for 30 to 60 minutes, depending on the location. Health professionals and patients are required to wear the recommended protection at all times [8]. Once the patch has been removed, the area is cleaned with the gel provided by the patch manufacturer and the patient is discharged.

### 2.6. Management of Treatment-Related Discomfort

As part of the treatment protocol, patients are asked to rate their actual discomfort level using the NPRS. This is reevaluated every 20 minutes for one-hour procedures and every 15 minutes for 30-minute procedures. Blood pressure is also taken after every pain rating. After the CP8% treatment has finished, patients are discharged and given two sheets of malleable frozen gel for home use. Instructions are also provided for extra treatment with nonsteroidal oral analgesics.

### 2.7. Efficacy

Treatment response (i.e., pain relief) was defined as a > 30% decrease in the NPRS from baseline to the posttreatment assessment at weeks 4–6. In cases in which the difference is exactly 30% or when the patient is unable to rate pain relief with the NPRS, then treatment efficacy is determined by asking the patient to estimate overall improvement and willingness to repeat treatment.

### 2.8. Statistical Analysis

The statistical analysis was performed with the VassarStats online software [19]. To check for differences in demographic data between responders and nonresponders, we performed a two sample *t*-test for independent samples for parametric values. *χ*^2^ was calculated for responders. Given that the total sample size was barely enough to perform the chi-squared test for some groups, the Fisher exact test was also calculated in these cases.

### 2.9. Difference in QTT for the Target and Control Areas

The patient sample was highly heterogenous due to variability in the following factors/conditions: time elapsed from injury to treatment; the use of multiple different concomitant medications; use of drugs acting on central nervous system (CNS) (i.e., antidepressants, psychoactive, or anticonvulsants) (see Table S4 in Supplementary Material available online at https://doi.org/10.1155/2017/7425907); different pain localizations involving one or several nerve territories. For these reasons, we were unable to compare out sample to normalized data reported elsewhere [20].

Since all patients in this sample had localized pain only, we used the contralateral asymptomatic healthy area as a control. The QTT profiles at the target PeLNP area were compared to the corresponding QTT profile at the contralateral area. As part of the routine treatment procedures, both WST and HPT values were assessed at baseline in the treatment (PeLNP site) area and at the asymptomatic contralateral site. Differences between the WST and HPT values in the target and control areas were considered not significant when there was a crossover between mean results (±1.96 SD) for the measurement on both areas; when this occurred, the painful area was considered to present normal thermal sensations. By contrast, if there was no crossover, we considered the WST (or HPT) for the treatment site to be significantly different (higher or lower) from the contralateral site.

## 3. Results

### 3.1. Patients

We initially identified a total of 65 patients diagnosed with PeLNP who had been treated with CP8% during the 2010–2013 period. Of these, 10 were excluded from the study for varying reasons: five were excluded because the contralateral area was not asymptomatic (contralateral pain in 5 cases and midline pain in 2 cases). Three other patients were excluded for the following reasons: one had an interventional pain procedure between treatment and assessment, another developed a recurrence requiring radiotherapy before the follow-up visit; and QTT data was not available in the third case. As a result, the final sample consisted of 55 patients.  Table 1 shows the demographic parameters organized according to responders and nonresponders. The mean age was 59 years old (range 32–82 years). There were no significant differences between the groups in terms of gender, age, DN4, or pretreatment NPRS scores. Of the 24 responders, the pain improvement lasted for > 6 months in 5 patients, where in 2 of them pain relief lasted for almost 12 months, while another patient fully recovered from the pain. For the other 19 patients (see Table 1), 7 of them had pain relief over 90 days, whereas 8 recovered between 60 and 90 days, and only 4 had recovery for less than 60 days or less (always more than 45 days). In all patients, the DN4 score was ≥ 4.

### 3.2. QTT Profile

**Figure 1:**
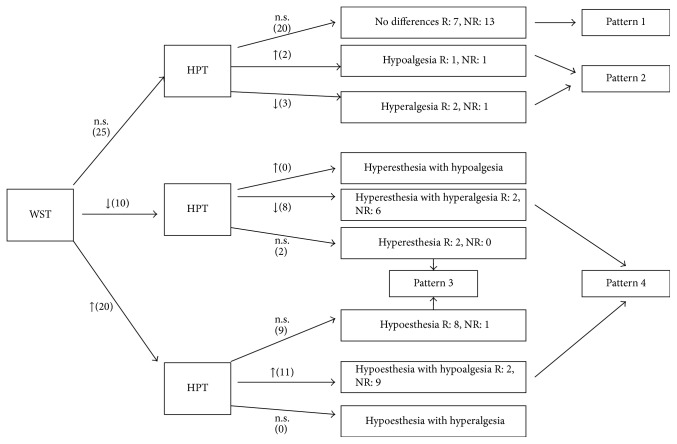
QTT profiles flowchart and responders/nonresponders to treatment. WST = warm sensation threshold. HPT = heat pain threshold. n.s. = not significant differences between painful and asymptomatic areas. ↑ = painful area with a significantly high difference versus the asymptomatic contralateral area. ↓ = painful area with a significantly low difference versus the asymptomatic contralateral area. In parenthesis (), number of patients with this profile. R = responders to treatment with capsaicin patch. NR = nonresponders to treatment with capsaicin patch. Clinical definitions such as hypoalgesia, hyperalgesia, hypoesthesia, and hyperesthesia are given to improve reading comprehension to understand the comparison versus the contralateral asymptomatic area; these QTT are not comparable to normalized published data.

Figure 1 is a flowchart of QTT profiles for WST and HPT values at the pain area versus the contralateral asymptomatic area. None of the patients presented a significantly high WST combined with a significantly low HTP, nor did any present the opposite (i.e., significantly low WST and significantly high HPT). Among the nonresponders, 4 patterns emerged.

*Pattern 1*. There were no significant differences in WST or HPT between the painful area and the control areal.

*Pattern 2*. WST is not significantly different between the control and target areas while the HPT value showed significant abnormal thermal sensations (either high or low) at the painful area.

*Pattern 3*. HPT presents no significant differences while the WST value shows significant abnormal thermal sensations (either high or low) in the painful area.

*Pattern 4*. Both WST and HPT values present significant differences between the two areas (both high or both low). Both WST and HPT show abnormal thermal sensations at the pain site.

**Figure 2:**
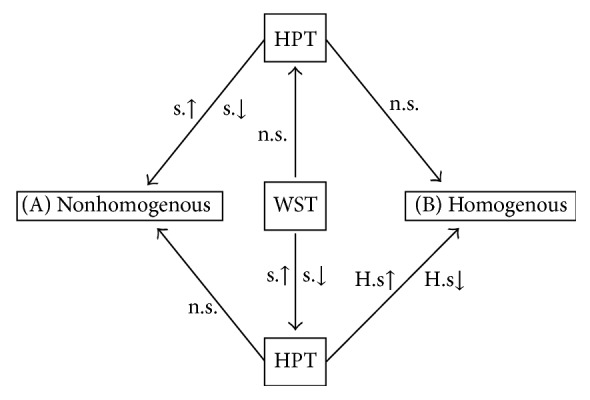
QTT profile groups identified after matching responder and nonresponders to treatment with capsaicin patch. WST: warm sensation threshold. HPT: heat pain threshold. n.s.: no significant difference between pain site and asymptomatic contralateral area for the thermal test. s.↑: the thermal test was significantly higher in the painful area versus the asymptomatic contralateral area. s.↓: the thermal test was significantly lower in the painful area versus the asymptomatic contralateral area. H.s↑/H.s↓: HPT for the painful area was significantly higher when WST was significantly higher or lower when WST was significantly lower than the asymptomatic contralateral area.

Two distinct groups were identified based on differences in QTT profiles for responders and nonresponders (Tables S1, S2, and S3). One group presented nonhomogenous WST and HPT results while the second group had homogenous WST and HPT results (Figure 2).

*(i) Homogenous Profile Group*. The first group presented homogenous WST and HPT profiles, defined as either the presence of significant differences in the same direction (both high or both low) in WST and HPT values between the PeLNP region and the asymptomatic contralateral area or no significant difference in these measures (both the treatment and control sites normal).

*(ii) Nonhomogenous Group*. The second group consisted of patients with nonhomogenous WST and HPT profiles, defined as the presence of significant differences between the PeLNP area and the contralateral site in only one (either WST or HPT) measure but not the other.

### 3.3. Predictors of Response to Treatment

#### 3.3.1. Demographics: Aetiology and Duration of Pain

There were no baseline differences between responders and nonresponders in terms of gender, age, DN4 scores, etiological diagnosis, or NPRS. There was a slightly larger number of females in the nonresponse group, but this was not statistically significant.

#### 3.3.2. Patterns and Responders

Table 2 shows the sensitivity, specificity, positive and negative predictive values, and positive and negative probability coefficients. As Table 2 shows, 24 patients improved after a single application of CP8% whereas 31 did not improve. The *χ*^2^ was 8.94 (*p* = 0.0028) and Fisher exact test (two tailed) was *p* = 0.0014 for patients who responded to treatment and had nonhomogenous WST and HPT values.

## 4. Discussion

At present, there are no reliable predictors of response to treatment with capsaicin for analgesia. Given that the application of capsaicin causes heat sensation, the aim of this study was to assess quantitative thermal testing (QTT) profiles in a group of patients with localized peripheral neuropathic pain (PeLNP) treated with topic capsaicin, to determine whether different QTT profiles could predict treatment outcomes. We did a retrospective analysis of 55 patients treated between 2010 and 2013 for PeLNP, where, as part of the routine treatment procedures, both warm sensation threshold (WST) and heat pain threshold (HPT) values were assessed at baseline in the treatment (PeLNP site) area and the asymptomatic contralateral site. From the analysis, two different groups were identified based on their QTT profile. The first group presented homogenous WST and HPT profiles, defined as either the presence of significant differences in the same direction (both high or both low) in WST and HPT values between the PeLNP region and the asymptomatic contralateral area or no significant difference in these measures (both the treatment and control sites normal). The second group consisted of patients with nonhomogenous WST and HPT profiles, defined as the presence of significant differences between the PeLNP area and the contralateral site in only one (either WST or HPT) measure but not the other. Most patients (27/31, 87.1%) with a homogenous profile were nonresponders. By contrast, more than half of the patients (13/24, 54.2%) with a nonhomogenous profile were responders (*p* = 0.0028). Although the reasons for this difference are not clear, we hypothesize patients with a partial loss of cutaneous nerve fibers or receptors are more likely to respond to treatment. By contrast, when severe nerve damage or normal cutaneous sensations are present, the pain is likely due to central sensitization and thus not responsive to capsaicin.

Potentially damaging mechanical, thermal, and chemical stimuli are detected by nerve endings called nociceptors found in the skin and other organs [21, 22]. The largest group of such nociceptors is the family of channels of the transient receptor potential (TRP) [23]. Within the TRP family, there are four different molecules (TRPV1, TRPV2, TRPV3, and TRPV4) that respond to different degrees of temperature increase, ranging from the perception of heat all the way up to harmful levels [24–26].

TRPV1 is a nonselective, ligand-dependent cationic channel that can be activated by a series of exogenous and endogenous physical and chemical stimuli, including temperatures above 43°C, low pH (in acid medium), endocannabinoids anandamide and N-arachidonyl-dopamine, or chemicals such as capsaicin (8-methyl-N-vanillyl-6-nonenamide) [27, 28], allowing the passage of different monovalent or divalent cations [29, 30]. TRPV1 is mainly expressed in peripheral nervous system (PNS) neurons such as the dorsal root ganglion (DRG) and in C and A*δ* sensory fibers [27, 28, 31]. In patients with neuropathic pain (NP), TRPV1 is expressed through the nociceptive pathway, from unmyelinated axons in the skin to the back of the spinal cord [22]. Transduction of the signal is achieved through the influx of sodium and calcium. In this way, the neurons expressing these receptors are depolarized [32, 33]. Many of the ligands that come into contact with TRPV1 possess synergistic effects, channel them integrally, and lead to a response or signal transduction [34].

In primary afferents, the nociceptors, upon activation, trigger the release of various peptides, including glutamate, neurokinin A, substance P, and the peptide related to the calcitonin gene in the dorsal horn (DH) of the spinal cord [35–38]. These neurotransmitters then trigger a series of signals that contribute to the activation of second-order sensory neurons, such as spinothalamic tract cells (STT) [39, 40] and other projection neurons. This causes the transmission of nociceptive information to the brain. In addition, signals are released that will activate GABAergic inhibitory interneurons of the DH [40, 41]. Together, the information that reaches the central nervous system (CNS) is interpreted as a burning pain or an itch, in addition to causing the peripheral release of proinflammatory substances that sensitize other neurons and subsequently give rise to other stimuli [32].

Multiple inflammatory stimuli increase the expression of TRPV1 and even increase the density of axons positive for TRPV1 [42–46]. A priori, this suggests that TRPV1 is involved in the pathogenesis of hyperalgesia and other pathological sensations. TRPV1 antiserum reduces thermal allodynia and hyperalgesia in diabetic mice [47]. In addition, TRPV1 antagonists reduce inflammation and NP [48]. Despite these known effects, the importance of TRPV1 in the pathogenesis of NP or peripheral neuropathies remains controversial [22]. It has been suggested that TRPV1 has an effect on the pathogenesis of nerve pain secondary to nerve damage [49]. However, some studies indicate that the TRPV1 receptor is not directly involved in the pathogenesis of PeNP [28, 50] and other studies suggest that the TRPV1 receptor does not contribute to the triggered hyperalgesia in situations of nerve injury [51]. In addition, numerous studies have reported conflicting data about the density of TRPV1 and the degree of nerve damage [52–57]. Taken altogether, these facts suggest that TRPV1 is not useful as a specific marker in cases of nerve injury [22]. However, more recent studies suggest that recipients of the same TRP family present patterns suggestive of an association between NP and injury, specifically, TRPV3 [58–60] and TRPV4 [46, 61]. Despite the aforementioned controversy, TRPV1 receptors continue to offer great therapeutic possibilities [62]. Given the ongoing research in this area, it seems likely that, in the near future, we will come to better understand the pharmacological potential of TRPV1.

Capsaicin is a well-known exogenous activator of TRPV1. Its pain relief effect is believed to be due to the activation of small diameter afferent nerve fibers and specialized DRG neurons that respond to many different noxious stimuli [63]. Capsaicin also mediates some actions of the endocannabinoid CB1 anandamide, which shares the same intracellular binding site as TRPV1 [64, 65]. Thus, capsaicin stimulates nociceptors and generates signal transmission to the brain, which interprets the signal as pain [40]. As a result, the application of capsaicin can desensitize the nerve terminals of nociceptors by inducing long-term desensitization after prolonged exposure. This desensitization allows the use of capsaicin as an analgesic, and its mechanism of action is based on the destruction of axons and, ultimately, of the DRG nociceptors [66].

Cutaneous injection of capsaicin activates the TRPV1 receptors, causing an influx of sodium and calcium ions into the cytoplasm of nociceptive neurons expressing that receptor [64]. At the capsaicin injection site, primary mechanical hyperalgesia and heat develop due to desensitization of the afferents containing TRPV1 receptors [67–69]. In a larger area surrounding the injection site, secondary mechanical hyperalgesia and allodynia take place. The primary effects of mechanical hyperalgesia and heat are believed to be attributable to sensitization of the primary afferent nociceptors [70]. However, at the site of secondary mechanical hyperalgesia and allodynia, the degree of excitability of first-order neurons is normal [40, 71, 72], implying that allodynia and secondary hyperalgesia depend on the CNS, such as STT. This effect is called central sensitization (CS) [33, 37, 40, 73–81]. This suggests that NP is maintained by second- or third-order neurons without a significant effect on the peripheral nerve afferents [82].

Previous studies [13] have found that patients can be grouped into various subgroups based on their response to treatment with CP8%. This finding suggests that response variability may be related to different pain mechanisms. One patient group in that study presented high variability in pain rating scores (i.e., mixed treatment response), whereas the low variability group were nonresponders. The authors suggested that the lack of response in this group could be due to long-term chronic pain and the presence of severe central plastic changes. By contrast, high variability in pain rating scores could be due to a more recent development of chronic pain status, as occurred in the CPSP group in our study. Given that the EMA authorised treatment in Europe for any condition with PeNP (except for diabetes), we included patients with a wide variety of etiological diagnoses (Table 1). Many of our patients had a diagnosis of CPSP, which is not surprising given that this diagnosis accounts for a large percentage of patients with PeNP [83, 84]. Our findings suggest that CPSP patients may be more responsive to CP8% than other groups. Martini et al. suggested that other factors or predictors related to the treatment of patients with chronic pain could enhance the ability to predict therapeutic efficacy. Specific patterns in the QST profile may represent specific phenotypes that have a greater or lesser probability of treatment response. Indeed, several authors have already shown that specific tests in the QST profile may represent a specific population that is more likely to respond to therapy. Eisenberg et al. [10] showed that the magnitude of heat pain thresholds predicts the magnitude of the decrease in pain intensity in response to oxycodone treatment (the greater the heat pain threshold, the greater the opioid effect; *R*2 = 0.17) in healthy volunteers. Yarnitsky et al. [85] showed in a sample of patients with painful diabetic neuropathy that patients with less efficient conditioned pain modulation have greater analgesic responses to duloxetine (*R*2 = 0.39).

The present study involves a cohort of patients treated as part of routine clinical practice at a single center. We retrospectively identified patients who had been treated with CP8% and who also had a pretreatment QTT profile test available. The main aim was to determine the existence (or not) of a QTT pattern that could help identify those patients likely to respond to CP8% treatment. The main limitation to this study is its retrospective design. Other limitations include the relatively small number of patients, which precluded the use of subgroup analysis. We found no differences in demographics (gender, age, and etiological diagnosis of PeNP) between responders and nonresponders. We assume that the demographic data represent a normal distribution although this cannot be verified. Another limitation is that the number of responders in our study was larger than expected in the context of the high NNT reported in other studies [86]. There are several possible reasons for this, particularly the calculation of the NNT itself [87]. Clinical trials were not compared to placebo, but rather to low-dose capsaicin (0.075%), which has been shown to be effective for the relief of NP [88]. The NNT could have been miscalculated, making it difficult to compare NNTs from a study that used a low-dose control to NNTs in studies using inert placebos [89]. Another reason for the high response rate in our study could be the inclusion of patients with variable etiological diagnoses for PeNP, such as CPSP. In addition, a placebo effect cannot be ruled out, especially given that controlled trials have described a placebo response rate ranging from 23% to 36% [7, 90]. Another limitation is that the medical team was not blinded, and this may have introduced bias into the patient assessment at follow-up.

We found that the clinical effects of CP8% were better in patients with nonhomogenous QTT profiles. These patients showed a significantly higher response rate than patients with homogenous QTT profiles. This difference may be due to incomplete nerve damage in these patients, leading to an imbalance in the sensitive inputs to second-order neurons from peripheral receptors and to the presence of ectopic discharges on nerve endings. If so, pain in these patients may be purely peripheral, with no additional CS mechanisms. Capsaicin application in these patients could eliminate the factor resulting in dysaesthesia when they activate the remaining TRPV1 receptors, desensitizing the nerve terminals of nociceptors by destroying the remaining axons and nociceptors.

By contrast, the group of patients with homogeneous QTT profiles had little or no clinical improvement. These patients either had no peripheral damage (normally functioning nociceptors) or may have had complete peripheral nerve damage (absent or nonfunctional nociceptors). When no peripheral nerve damage is present, there should not be any receptor loss and thus there should be no differences in WST/HPT values between the painful area and the asymptomatic contralateral area. The opposite should also be true: in the case of complete peripheral nerve damage, nearly complete loss of receptors is to be expected, meaning that both warmth and heat sensations are likely to significantly differ between the painful and control areas. Thus, in both of these cases (i.e., complete peripheral nerve damage and no damage at all), the QTT tests should be homogenous, with capsaicin therapy unlikely to be efficacious in either of these two groups. Indeed, our results point in this direction. Pain in these patients could be due to CS mechanisms, with inputs multiplied at the DH as explained above; that is, the origin of the pain in these patients is probably less peripheral and more central. For this reason, the capsaicin is less effective in providing pain relief.

In the 3rd pattern identified (i.e., no significant differences in the HPT but significant differences in the WST) the hypoesthesia (high WST only) subgroup presented a better response rate (8 responders versus only 1 nonresponder). Although we were unable to perform a subgroup analysis due to the small sample size, these findings support the hypothesis developed by Malmberg et al. [6], who argued that the foremost psychophysical manifestation of topical capsaicin treatment is a reduced sensitivity to heat stimuli. This is the expression of an elevated-warmth detection threshold, corresponding to a loss of cutaneous sensory nerve fibers.

Overall, and within the aforementioned study limitations, our results seem to confirm previous reports. Höper et al. [12] evaluated sensory neuropathic abnormalities (painDETECT questionnaire), finding that the presence of burning and pressure-evoked pain was weakly associated with treatment response. They argued that thermal hyperalgesia is difficult to interpret and thus cannot serve as a predictor of response, which could be ascribed to the fact that the painDETECT questionnaire does not distinguish between cold and heat-evoked pain. Consequently, they concluded that data on heat-evoked pain, which is very likely TRPV1 receptor-dependent, would be preferable to predict treatment response to CP8%. Edwards et al. [91] found that the HPT values in the affected area and at the corresponding contralateral side predicted the effect of morphine and methadone on pain in patients with PHN. We found a similar association between certain QTT profiles and capsaicin response when we compared the pain site to the contralateral asymptomatic area. Gustorff et al. [11] identified potential differences in the sensory profiles—particularly the pressure pain threshold and degree of allodynia—of patients with PeNP who responded to CP8% and those who did not. In that study, the authors were unable to find warm hyperesthesia of heat hyperalgesia in responders, and they found similar WST/HPT profiles at baseline for both responders and nonresponders. By contrast, we looked at QTT profiles from a different point of view, using the contralateral asymptomatic area as the only control reference.

Despite the findings described above, it is possible that unilateral PeLNP may be associated with bilateral changes in PNS [92]. Thresholds measured on the contralateral side in PeLNP patients may not represent basal pain sensitivity. For this reason, we considered the homogeneity of QTT profiles rather than clinical symptoms (i.e., heat hyperalgesia). Martini et al. [13] found one group of patients with PeLNP whose pain could be attributed to a rigid and fully manifested chronic pain process with severe CS plastic changes that were unresponsive to therapy. In our study, we were aware that central or bilateral peripheral changes could occur—even though our sample consisted of patients with PeLNP—and for this reason we considered differences between the treatment and contralateral areas to be significant only when there was no crossover between mean results (±2 SD). Using these criteria, two patterns were found for responders and nonresponders when we examined profile homogeneity. For instance, a significantly differently low HPT (i.e., heat hyperalgesia) with no significant difference in WST was considered nonhomogeneous. By contrast, if the WST was also significantly different between the control and treatment areas, then the QTT profile was considered homogenous. Based on our results, it appears that patients who show a nonhomogenous profile in terms of WST and HPT values are significantly more likely to respond to capsaicin treatment, probably due to the presence of incomplete nerve damage. Nevertheless, these findings need to be confirmed in a prospective controlled blinded study, preferably with a large sample to enable subgroup analysis to better identify the QTT profile of responders.

## Supplementary Material

Tables S1, S2 and S3 represent the distribution of quantitative thermal tests for patients for different etiologies. Table S1 represent patients who suffered from Post-Herpetic Neuralgia. Table S2 represent patients who had chronic post-surgical pain. Table S3 represent other etiologies. All tables, patients are grouped for Responders and Non-Responders (see righthand column). Secondly to it, patients were grouped for homogeneity (see second to the right, consistency column) between results for warm sensation threshold and heat pain threshold for the painful area compared to the control asymptomatic area. From the tables it can be appreciated that non-responders are mostly present within the non-homogeneous results, being the non-homogeneous the ones with more responders in it.

## Figures and Tables

**Table 1:** Demographic data according to treatment response.

Variable	Nonresponders	Responders	*p* value
Gender (female/male ratio)	19/12	13/11	0.28
Age (mean)	60.26 (14.25)	58.42 (13.28)	0.62
DN4 score	5.90 (1.37)	5.39 (1.29)	0.24
Pretreatment NPRS	7.21 (1.20)	6.89 (1.75)	0.79
Posttreatment NPRS	7.08 (1.29)	2.92 (1.38)	n.a.
Duration of improvement, months (mean)	b	72.89 (24.68)^a^	n.a.
Type of PeNP			0.88^c^
CPSP	18	13	
PHN	12	8	
Posttrauma	0	1	
CRPS	1	2	

DN4 = *Douleur Neuropathique 4 Questionnaire*. NPRS = numerical pain rating scale (0–10). CPSP = chronic postsurgical pain. PHN = postherpetic neuralgia. CRPS = complex regional pain syndrome type I. n.a. = not applicable. Gender and type of PeNP for the whole patient sample. For the others, numbers are given as means. Standard deviation in parenthesis. a = calculated for 19 patients. The other 5 were excluded due to reporting > 6 months of improvement. b = nonresponders had no days of improvement, most of whom reported no improvement at all. c = posttrauma and CRPS were not taken into account for the analysis due to the small number of patients.

**Table 2:** Contingency table.

	Total	No improvement	Improvement	
PPV = 76%	17	4	13^a^	Nonhomogenous (WST ≠ HPT)
NPV = 71%	38	27	11	Homogenous (WST = HPT)
	55	31	24	Total
		E = 87%	S = 54%	
		NLR = 0.52	PLR = 4.2	

Contingency table for the QTT profiles and patterns observed as a predictor of improvement after application of the capsaicin 8% patch. WST ≠ HPT: nonhomogenous results for warm sensation threshold and heat pain threshold in the peripheral neuropathic pain area versus the contralateral control area, with one measurement (either WST or HPT) showing a significant difference between the control and treatment areas while the other measurement (either WST or HPT) is not significantly different between the two sites. WST = HTP: both warm sensation threshold and heat pain threshold had homogenous test results in the pain and control areas: both were either significantly higher or lower, or neither was significantly different. S: sensibility. E: specificity. PPV: positive predictive value. NPV: negative predictive value. PLR: Positive Likelihood Ratio. NLR: Negative Likelihood Ratio. ^a^*χ*^2^ = 8.94 (*p* = 0.0028). Fisher exact test *p* = 0.0014 (two tailed).

## Additional Points

*What is already known about this topic?* (i) There are no reliable predictors of response to treatment for localized neuropathic pain. (ii) Although some studies have attempted to identify predictors of treatment response, none have proven reliable.

*What does this study add?* (i) Quantitative thermal tests may help to predict which patients will respond to capsaicin patch treatment. (ii) Patients with partial damage to peripheral nerves may respond better to the mechanism of action of topical capsaicin than patients with completely damaged or undamaged peripheral nerves.
