# Supplementary material for: Quantitative Thermal Testing Profiles as a Predictor of Treatment Response to Topical Capsaicin in Patients with Localized Neuropathic Pain
Source: Pain Res Treat. 2017 Feb 21;2017:7425907. doi: 10.1155/2017/7425907 (PMC5339491; doi:10.1155/2017/7425907)
Supplement: Supplementary file 1 — Tables S1, S2 and S3 represent the distribution of quantitative thermal tests for patients for different etiologies. Table S1 represent patients who suffered from Post-Herpetic Neuralgia. Table S2 represent patients who had chronic post-surgical pain. Table S3 represent other etiologies. All tables, patients are grouped for Responders and Non-Responders (see righthand column). Secondly to it, patients were grouped for homogeneity (see second to the right, consistency column) between results for warm sensation threshold and heat pain threshold for the painful area compared to the control asymptomatic area. From the tables it can be appreciated that non-responders are mostly present within the non-homogeneous results, being the non-homogeneous the ones with more responders in it. [file 7425907.f1.pdf]

Table S1: individual results for Warm/Heat pain sensations and response to treatment. Results for patients with Postherpetic Neuralgia (PHN).

Patient ID	Cause of PeLNP	Differences in WST	Differences in HPT	Consistency	Response to CP8%
11TERM	PHN	Yes	yes	yes	no
14TERM	PHN	Yes	yes	yes	no
15TERM	PHN	Yes	yes	yes	no
18TERM	PHN	Yes	yes	yes	no
33TERM	PHN	Yes	yes	yes	no
37TERM	PHN	Yes	yes	yes	no
19TERM	PHN	No	no	yes	no
20TERM	PHN	No	no	yes	no
21TERM	PHN	No	no	yes	no
17TERM	PHN	No	no	yes	no
70TERM	PHN	Yes	no	no	no
25TERM	PHN	No	yes	no	no
05TERM	PHN	No	yes	no	yes
38TERM	PHN	No	yes	no	yes
36TERM	PHN	Yes	no	no	yes
39TERM	PHN	Yes	no	no	yes
64TERM	PHN	Yes	no	no	yes
08TERM	PHN	No	no	yes	yes
59TERM	PHN	No	No	yes	yes
58TERM	PHN	Yes	yes	yes	yes

Table S2: individual results for Warm/Heat pain sensations and response to treatment.Patients with Chronic Postsurgical Pain (CPSP) neuropathic pain.

Patient ID	Cause of PeLNP	Differences in WST	Differences in HPT	Consistency	Response to CP8%
69TERM	CPSP	Yes	Yes	yes	no
13TERM	CPSP	Yes	Yes	yes	no
16TERM	CPSP	Yes	Yes	yes	no
24TERM	CPSP	Yes	Yes	yes	no
31TERM	CPSP	Yes	Yes	yes	no
32TERM	CPSP	Yes	Yes	yes	no
35TERM	CPSP	Yes	Yes	yes	no
40TERM	CPSP	Yes	Yes	yes	no
23TERM	CPSP	no	No	yes	no
26TERM	CPSP	no	No	yes	no
27TERM	CPSP	no	No	yes	no
28TERM	CPSP	no	No	yes	no
29TERM	CPSP	no	No	yes	no
30TERM	CPSP	no	No	yes	no
34TERM	CPSP	no	No	yes	no
42TERM	CPSP	no	No	yes	no
12TERM	CPSP	yes	No	no	no
63TERM	CPSP	no	Yes	no	no
02TERM	CPSP	no	Yes	no	yes
01TERM	CPSP	yes	No	no	yes
09TERM	CPSP	yes	No	no	yes
10TERM	CPSP	yes	No	no	yes
51TERM	CPSP	yes	No	no	yes
62TERM	CPSP	yes	NO	no	yes
56TERM	CPSP	yes	No	no	yes
57TERM	CPSP	yes	Yes	yes	yes
03TERM	CPSP	yes	Yes	yes	yes
41TERM	CPSP	no	No	yes	yes
55TERM	CPSP	no	No	yes	yes
06TERM	CPSP	no	No	yes	yes
65TERM	CPSP	no	No	yes	yes

Table S3: individual results for Warm/Heat pain sensations and response to treatment.Patients with other causes of Neuropathic pain.

Patient ID	Cause of PeLNP	Differences in WST	Differences in HPT	Consistency	Response to CP8%
04TERM	traumatic	yes	No	no	yes
07TERM	CRPS	yes	yes	yes	yes
52TERM	CPRS	NO	NO	yes	yes
22TERM	CPRS	no	No	yes	no

Table S4. Concomitant medication.

Patient ID	Cause of PeLNP	Drugs at the time of the patch
69TERM	CPSP	Paracetamol/acetaminophen
13TERM	CPSP	Carbamazepine
16TERM	CPSP	Pregabalin. Paracetamol. Ibuprofen. Fentanyl transdermal patch
24TERM	CPSP	Lidocaine patch
31TERM	CPSP	Lidocaine patch, Paracetamol/acetaminophen
32TERM	CPSP	Gabapentine, Paracetamol/acetaminophen
35TERM	CPSP	Paracetamol/acetaminophen. Ibuprofen. Buprenorphine
40TERM	CPSP	Metamizole. Lidocaine patch
23TERM	CPSP	Lidocaine patch
26TERM	CPSP	Lidocaine patch. Clonazepam
27TERM	CPSP	Paracetamol/acetaminophen. Tramadol. Pregabalin
28TERM	CPSP	Paracetamol/acetaminophen. Tramadol. Pregabalin
29TERM	CPSP	Tramadol. Pregabalin. Duloxetine
30TERM	CPSP	Paracetamol/acetaminophen
34TERM	CPSP	Tapentadol
42TERM	CPSP	Pregabalin
12TERM	CPSP	Gabapentine
63TERM	CPSP	Gabapentine
02TERM	CPSP	Paracetamol/acetaminophen
01TERM	CPSP	Lidocaine patch. Alprazolam
09TERM	CPSP	Paracetamol/acetaminophen. Tramadol. Pregabalin
10TERM	CPSP	Metamizole
51TERM	CPSP	Methadone. Metamizole
62TERM	CPSP	Paracetamol/acetaminophen
56TERM	CPSP	Gabapentine. Tramadol. Lidocaine patch
57TERM	CPSP	Gabapentine
03TERM	CPSP	Metamizole. Ibuprofen
41TERM	CPSP	Paracetamol/acetaminophen. Pregabalin
55TERM	CPSP	Paracetamol/acetaminophen
06TERM	CPSP	Nortriptyline
04TERM	Traumatic	Paracetamol/acetaminophen. Lidocaine patch
11TERM	PHN	Tapentadol. Pregabalin. Paracetamol/acetaminophen
14TERM	PHN	Lidocaine patch
15TERM	PHN	Lidocaine patch
18TERM	PHN	Pregabalin. Paracetamol/acetaminophen. Tramadol

33TERM	PHN	Amitriptyline
37TERM	PHN	Paracetamol/acetaminophen.
19TERM	PHN	Amitriptyline. Gabapentine
20TERM	PHN	Oxycodone/naloxone. Paracetamol/acetaminophen.
17TERM	PHN	Amitriptyline. Gabapentine
70TERM	PHN	Mirtazapine. Pregabalin
25TERM	PHN	Oxycodone/naloxone. Gabapentine
05TERM	PHN	Clonazepam. Paracetamol/acetaminophen.
38TERM	PHN	Paracetamol/acetaminophen. Ibuprofen
36TERM	PHN	Pregabalin. Lidocaine patch
39TERM	PHN	Metamizole. Gabapentine. Lidocaine patch
64TERM	PHN	Amitriptyline
08TERM	PHN	Paracetamol/acetaminophen. Lidocaine patch
59TERM	PHN	Gabapentine
58TERM	PHN	Lidocaine patch
52TERM	CRPS	Paracetamol/acetaminophen. Ibuprofen
07TERM	CRPS	Oxycodone/naloxone. Paracetamol/acetaminophen.
22TERM	CRPS	Carbamazepine

Table S1:
Colors are used to help identify groups of consistency and responders to treatment. PeLNP= Peripheral Localised Neuropathic Pain. WST=Warm Sensation Threshold. HPT=Heat Pain Threshold. CP8%=8% Capsaicin patch.

Table S2:
Colors are used to help identify groups of consistency and responders to treatment. PeLNP= Peripheral Localised Neuropathic Pain. WST=Warm Sensation Threshold. HPT=Heat Pain Threshold. CP8%=8% Capsaicin patch.

Table S3:
Colors are used to help identify groups of consistency and responders to treatment. PeLNP= Peripheral Localised Neuropathic Pain. WST=Warm Sensation Threshold. HPT=Heat Pain Threshold. CP8%=8% Capsaicin patch.

Table S4. Concomitant medication. In this table we can see how the subjects that make up the study are treated with one or more drugs.
